# Applications of Modified Mesenchymal Stem Cells as Targeted Systems against Tumor Cells

**DOI:** 10.3390/ijms25147791

**Published:** 2024-07-16

**Authors:** Elsa N. Garza Treviño, Adriana G. Quiroz Reyes, Paulina Delgado Gonzalez, Juan Antonio Rojas Murillo, Jose Francisco Islas, Santiago Saavedra Alonso, Carlos A. Gonzalez Villarreal

**Affiliations:** 1 Laboratorio de Terapia Celular, Departamento de Bioquímica y Medicina Molecular, Facultad de Medicina, Universidad Autónoma de Nuevo León, Av. Dr. José Eleuterio González 235, Monterrey 64460, Nuevo León, Mexico; elsa.garzatr@uanl.edu.mx (E.N.G.T.); guadalupe.quirozrys@uanl.edu.mx (A.G.Q.R.); paulina.delgadogn@uanl.edu.mx (P.D.G.); juan.rojasmrll@uanl.edu.mx (J.A.R.M.); jislas.me0117@uanl.edu.mx (J.F.I.); 2 Departamento de Ciencias Básicas, Vicerrectoría de Ciencias de la Salud, Universidad de Monterrey, Ignacio Morones Prieto 4500, Jesus M. Garza, San Pedro Garza García 66238, Nuevo León, Mexico

**Keywords:** cancer, mesenchymal stem cell, cell therapy, exosomes

## Abstract

Combined gene and cell therapy are promising strategies for cancer treatment. Given the complexity of cancer, several approaches are actively studied to fight this disease. Using mesenchymal stem cells (MSCs) has demonstrated dual antitumor and protumor effects as they exert massive immune/regulatory effects on the tissue microenvironment. MSCs have been widely investigated to exploit their antitumor target delivery system. They can be genetically modified to overexpress genes and selectively or more efficiently eliminate tumor cells. Current approaches tend to produce more effective and safer therapies using MSCs or derivatives; however, the effect achieved by engineered MSCs in solid tumors is still limited and depends on several factors such as the cell source, transgene, and tumor target. This review describes the progress of gene and cell therapy focused on MSCs as a cornerstone against solid tumors, addressing the different MSC-engineering methods that have been approached over decades of research. Furthermore, we summarize the main objectives of engineered MSCs against the most common cancers and discuss the challenges, limitations, risks, and advantages of targeted treatments combined with conventional ones.

## 1. Introduction

Mesenchymal stem cells (MSCs) are a type of multipotent cell found in various body tissues [1], including bone marrow [2], adipose tissue [3], the umbilical cord [4] and other connective tissues. In addition to their ability to differentiate into a range of cell types, such as osteoblasts, chondrocytes, adipocytes, and muscle cells [1], MSCs also exhibit immunomodulatory and anti-inflammatory properties that make them extremely valuable for research and clinical application in regenerative therapies and the treatment of various diseases [5]. MSCs have been studied in vitro and in vivo as [6] they possess several characteristics that make them an excellent gene delivery vehicle. For example, they can be easily transduced by different methods [7] and expanded in vitro to generate many modified cells, enhancing the production of proteins (cytokines, growth factors, such as IL-1α, IL−1β, IL−4, IL-5, IL-6, IL-12, MIP-2, TNF−α, and IFN-γ or target proteins such as TRAIL, Myc, HER2) or therapeutic compounds such as doxorubicin, paclitaxel, 5FU, Gemcitabine, Sorafenib, Curcumin, etc.) [6]. On the other hand, MSCs can migrate to specific sites, such as areas of inflammation or injury, and integrate into these tissues [8]. In the field of cancer, MSCs have been shown to possess a tumor-homing ability, which is mediated by chemokines secreted by tumors [9]. This fact makes them a good option for biodirected anticancer therapies and a particularly useful alternative on tumor spread/metastasis.

The introduction of therapeutic genes, like suicide genes, tumor-suppressor genes, pro-apoptotic genes, gene-encoding immune activation genes, and gene-encoding cytokines [10] into MSCs has led to the development of new therapies that have been successful at the inhibition tumor growth, activating the immune response and inducing apoptosis. For example, *Her2*, *APC*, *p53*, *Myc*, *Bcl-2*, *KRAS* are oncogenes or mutations that occur frequently in human cancer and are often associated with aggressive disease and poor prognosis

However, it is important to identify specific targets in cancer cells to which MSCs will be directed. These must be specific to avoid damaging adjacent tissues. Several therapeutic targets have been reported to which genetically modified MSC therapy can be directed. Some therapeutic targets that are used in solid tumors to deliver genes induce cytokine secretion, or activate/inhibit cell receptors, are shown in Table 1.

The ability of MSCs to be genetically modified in vitro is their primary characteristic as cellular carriers for gene therapy [52]. Viral vectors (adenoviral, lentiviral, retroviral, adeno-associated virus), non-viral vectors (plasmids, liposomes), and chemical methods (nanoparticles) have been used to insert therapeutic genes into MSCs [53]. Each of these methods has advantages and disadvantages. For example, viral methods are more efficient in introducing transgenes into MSCs. They lead to stable gene expression, but their clinical applications are limited because of oncogenic transformation and the induction of immune responses [10,52].

On the other hand, non-viral methods have low efficiency and lead to transient expression of the desired transgene but are deemed safer for human application as some undesirable side effects, such as cancerous changes brought on by incorrect gene modification [54]. Loading MSCs with nanoparticle drugs increases efficacy and externally moderates targeting [54]. The latent risk of cells turning malignant or triggering immune reactions has led to the investigation of cell-free approaches such as microvesicles (exosomes).

The method of choice to genetically modify MSCs will depend on the type of therapeutic approach used and the type of cancer treated. In this review, we analyze the different MSC gene modification methods, their advantages and disadvantages, and their applications in targeting solid carcinogenic tumors, with an emphasis on breast, lung, and colon cancer.

## 2. Plasmid-Based Genetic Modification of Mesenchymal Stem Cells

Plasmid-based gene therapy has been attempted to correct individual genetic disorders. The first approved human gene therapy clinical trial was conducted in 1990, aiming to introduce a gene-replacing adenosine deaminase deficiency [55]. Since then, hundreds of gene therapy protocols have been approved or implemented.

The predominant DNA-based vectors used in cancer gene therapy and DNA vaccination are plasmids [56]. These are circular, double-stranded DNA constructs ranging in size from <1000 to >200,000 base pairs [57]. Originally obtained from bacteria, plasmids undergo vertical transmission during bacterial cell division, replicating multiple times within the resulting identical daughter cells.

Despite plasmid transfection having been widely used on gene therapy as a non-viral method, MSCs are known to be complicated to transfect by conventional methods, such as cationic lipids or non-liposomal lipids [58], showing low efficiency and low protein production yield, thus possessing an important limitation to carry transgenes; therefore, other methods have displaced plasmid transfection (such as viral vectors). However, inherent risks of viral vectors, mainly random integration into the host genome and possible presentation of viral antigens, are important drawbacks that plasmid vectors do not present [59].

**Figure 1:**
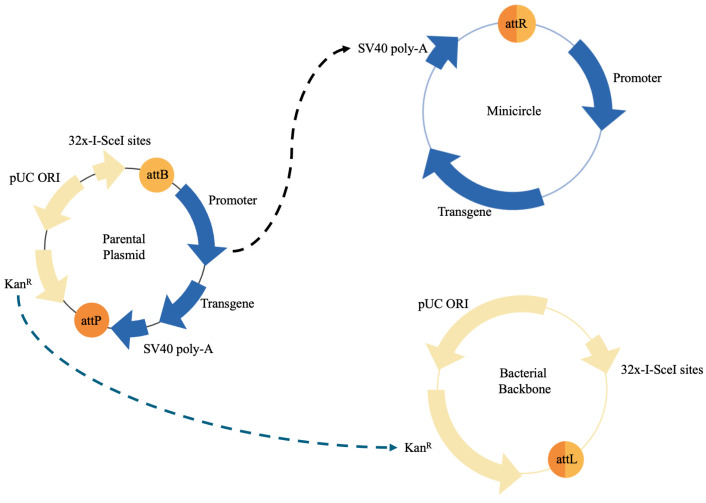
Plasmid and Minicircle System Components.

Recent modifications have been made to plasmids that can carry and express therapeutic genes more efficiently and safely than conventional plasmids. These enhanced versions of plasmids are called “minicircle DNA” (mcDNAs) [60] (Figure 1). Their structure is more compact than plasmids due to the absence of non-essential or redundant sequences such as regulatory elements and antibiotic selection genes. These modifications make mcDNAs less immunogenic and more stable than traditional plasmids [61]. Florian et al. achieved a 3.7-fold increase in angiopoietin-1 (ANPT1) expression with minicircle plasmids versus a conventional expression plasmid vector (pVAX-CMV-1) using nuclear targeted electroporation [62]. On the other hand, more efficient transfection methods have been reported; Khei Ho et al. reported 80% transfection efficiency using lineal polyethylenimine which successfully expressed cytosine deaminase::uracil phosphoribosyltransferase (CDy::UPRT) with positive results against breast, glioma and gastric cell lines [63]. A Phase I trial examines the side effects and optimal dose of a multiantigen DNA plasmid-based (CD105/Yb-1/SOX2/CDH3/MDM2-polyepitope) DNA vaccine for treating patients with HER2-negative, stage III-IV breast cancer (NCT02157051) [64]. This type of vaccine targets immunogenic proteins expressed in breast cancer stem cells, which are often resistant to treatment and capable of metastasis. DNA-based vaccines may help the body develop an effective immune response to eliminate tumor cells. Also, it was recently reported that the transfection of recombinant plasmid encoding CTNF-α to MSCs produces anti-tumoral peptides [65]. Another example is SGT-53, which is a complex composed of a wild type p53 gene (plasmid DNA) encapsulated in a liposome that is targeted to tumor cells by means of an anti-transferrin receptor single-chain antibody fragment (TfRscFv) attached to the outside of the liposome [66,67,68]. Pre-clinical studies have indicated that SGT-53 could sensitize tumors to the effects of radiation/chemotherapy [67]. Another study reported that hAMSCs genetically engineered with polymeric nanoparticles containing BMP4 plasmid DNA (BMP4/NP-hAMSCs) secrete the BMP4 growth factor while retaining their multipotency and preserving their migration and invasion capabilities. This study demonstrated that in vivo administration of hAMSCs genetically engineered with PBAE nanoparticles has a significant therapeutic effect in a human malignant glioma model [66].

## 3. Exosomes

Microvesicles or exosomes are produced by most cells, but stem cells rely heavily on communication through exosome secretion. Exosomes are round or cup-shaped lipid bilayer vesicles with a diameter of 30–100 nm, a density of 1.13–1.19 g/mL [69], containing several biomolecules, mainly proteins and RNA (mRNA and iRNA), that can orchestrate a myriad of effects on surrounding cells.

Exosomes are derived from multivesicular bodies and released into the extracellular matrix. They communicate with other cells by fusing with the membrane through endocytosis [70] and are responsible for crosstalk between MSC and other cells, playing a critical role in cancer behavior.

Tumors have been described as “wounds that do not heal”; thus, they are targeted by MSC homing [71]. Within the tumor, MSC exosomes exert various effects: acting as promoters or suppressors of mechanisms involved in cell growth, apoptosis, drug sensitivity or resistance, and angiogenesis through different pathways. The main pathways involved include AKT, ERK, Hedgehog, WNT, and CaM-Ks/Raf/MEK/ERK [72,73]. The specific mechanisms depend on the cellular origin cellular and type of cancer; however, such mechanisms are regulated by several miRNAs. For example, Allabhaneni and collaborators observed that exosomes released by serum-derived hMSCs could induce breast cell proliferation by transferring miRNA-21 and miR-34a [74]. In another study, miR-221 was identified as a highly specific microRNA in exosomes derived from gastric cancer tissue MSCs; these exosomes facilitated the transfer of functional miR-221 to gastric cancer cells, promoting their proliferation and migration [75]. Conversely, Roccaro et al. [76] found that the microRNA content in exosomes differed between normal bone marrow-derived MSCs (BM-MSCs) and multiple myeloma (MM) BM-MSCs. Due to their high content of the tumor suppressor miR-15a, exosomes derived from MM BM-MSCs promoted MM tumor growth, while normal BM-MSC exosomes inhibited the growth of MM cells.

Exosomes represent a promising cell-free approach to deliver drugs or other biomolecules for therapeutic purposes as they are easier to produce [77,78]. They show a long circulating half-life, a small size and high plasticity to pass through tissues [79], no ethical issues, no immunogenicity (they pose virtually no risk of triggering an immune reaction [80]), and most importantly, their cargo can be modified and produced in high concentrations; however, there are still setbacks to overcome such as carrier separation, purification, drug loading, and efficient targeting [81]; furthermore, although exosomes are very stable vesicles, there can be inconsistencies in production. Lastly, there are no guidelines for therapeutic agents [82].

Anticancer drugs, prodrugs, or proteins can be loaded in exosomes [83]. MSCs’ capacity to secrete exosomes (greater than other cells) results in a synergistic anti-cancer approach with promising results delivering drugs such as doxorubicin [84] and paclitaxel [85] on colon and breast cancer models, respectively. Recent research has also focused on developing more efficient cargo delivery, taking advantage of the small size and permeability of exosomes to enhance its targeting system [86], as shown in Table 2.

It is worth mentioning that the majority of the clinical trials using MSC exosomes are oriented to regenerative medicine approaches or chronic inflammatory diseases; in addition, most cancer-oriented work is in a preclinical phase; however, some notable studies have already escalated to clinical trials. Briefly: the Phase I, clinical trial NCT03608631, studies the dose and efficacy of MSC-EXO loaded with siRNAs (iexosomes) against patients with pancreatic cancer carrying mutant KrasG12D [97]. The clinical trial, NCT06245746, explores the use of UCMSC-EXO (umbilical cord-derived MSCs) to mitigate common myelosuppression induced by chemotherapy in myeloid leukemia patients after achieving remission (thus, it is not proper cancer therapy) [98]. This approach enables precise and targeted delivery of genetic material to specific cells, offering diverse applications in research and medicine.

## 4. Use and Applications of Viral Vectors by Modifying MSCs against Tumor Cells

MSCs have high recombinant virus infection efficiency, expressing optimal target protein concentrations, therefore making them excellent carriers for gene therapy. There are different viral transduction platforms. The transduction process is characterized by the transfer of genes into target cells by viral vectors. A viral vector consists of three components: (1) the protein capsid and/or envelope that encapsidates the genetic material; (2) the transgene of interest, which, when expressed in cells, confers the desired effect; and (3) the “regulatory cassette,” the combined enhancer/promoter/auxiliary elements that control the stable or transient somatic expression of the transgene as an episome or a chromosomal integrant [99].

The most prevalent viral vectors that have been extensively used for MSC transduction are based on adenovirus (Ad), adeno-associated virus vectors (AVVs), and lentivirus. Virus vectors have different properties, such as capacity insert size, cell/tissue tropism, and the ability to infect dividing cells, as shown in Table 3.

Many groups have studied MSCs as a viral vector-based delivery system. Some are described as follows. Oncolytic virus highly eliminates cancer cells; however, optimal delivery into the tumor stroma is crucial to achieve a significant effect. MSCs, as a delivery platform for oncolytic adenovirus, have shown better antitumor effects and increased survival in xenograft models of solid tumors [109]. Different research groups have demonstrated that MSCs carry oncolytic adenovirus-arrested tumor growth and metastasis development. MSCs delivering oncolytic adenovirus ICOVIR5 and CRAd5/F11 in a mouse model of lung and colorectal cancer inhibited tumors by activation of T cell migration to the tumor site [110]. Changes in the oncolytic adenovirus structure, such as removing the antiapoptotic gene E1B19K and replacing it with TRAIL gen, decreased the tumor size and reduced proliferation and cancer stem cell markers Ki67 and CD24 while increasing caspase activation [111].

Adenovirus serotype 5 (Ad5) is a frequently used platform of recombinant adenoviruses. MSCs have been modified with Ad5 to produce the oncolytic adenovirus, which reduces lung cancer tumor growth in A549 xenograft mouse models. The addition of regulatory systems based on doxycycline resistance, such as E1B55K, increased viral production and oncolytic virus release at the tumor site, inducing apoptosis via p53 accumulation [112]. In vitro studies of breast cancer showed that human MSC-Ad5/3.CXCR4 cells induce oncolysis in MDA-MB-231 cells at an MOI of 1000 at day 3. Moreover, they reduced lung metastasis in treated mice [113]. MSCs transduced with adenoviral vectors for CXCL1 expression inhibited the development of lung metastasis and improved mouse survival in tumor-bearing mice induced by melanoma (B16F10) and colon cancer (C26) cell lines [114]. Adenoviral transduction of bone marrow-derived MSCs for pigment epithelium-derived factor (PEDF) expression was studied as a treatment for Lewis lung carcinoma (LLC). The systemic administration of PEDF MSC reduced the growth of LLC tumors and prolonged mouse survival. Apoptosis was confirmed by immunohistochemistry, while a decrease in microvessel density was observed [115]. In addition, MSCs loaded with oncolytic adenovirus inhibited tumor growth in breast cancer murine models due to several factors, such as oncolytic viruses replicated within cancer cells, leading to cell destruction (lysis) and ultimately reducing tumor growth and improving survival rates in lung and breast cancer animal models [116]. Adenoviral transduction of MSC for TRAIL expression blocks tumor growth in a xenograft mouse model of the A549 lung cancer cell line [117]. MSCs modified with the AdEasy Adenoviral Vector System for expressing IFN-β inhibited the proliferation of breast cancer cells MDA 231 when administrated in situ [118].

On the other hand, lentiviral transduction is integrative to the transgene, providing permanent and stable expression. MSCs transduced for TRAIL expression induce apoptosis in the TRAIL-resistant colorectal cancer cell line HT29 and inhibit xenograft growth. Moreover, combination with 5-FU or oxaliplatin chemotherapy sensitizes TRAIL-MSCs resistance in vitro. The proposed mechanism is through mitochondrial disruption [119]. In addition, pre-treatment of the colorectal cancer cell line Caco-2 with oxaliplatin increases soluble TRAIL cytotoxic and pro-apoptotic activity [120]. TRAIL-expressing MSCs generate apoptosis of lung cancer cell lines and reduce metastasis in 40% of mice [121].

The administration of lentiviral-transduced MSCs co-expressing TNF-α and CD40L increased mouse survival in a breast tumor model, optimizing the antitumor immunity response in the presence of dendritic cells [122]. Human umbilical cord-derived MSCs genetically modified with lentivirus to deliver ISZ-sTRAIL-induced apoptosis and reduced tumor growth in a xenograft mouse model of lung cancer that had migrated to the tumor site by the MCP-1/CCR2 axis [123]. The systemic administration of lentiviral-modified MSCs expressing lipocalin 2 reduces liver metastasis by downregulating vascular endothelial growth factors in murine colon cancer with the SW48 cell line [124]. Apoptin-modified MSCs with lentivirus produce apoptosis via caspase-3 activation and, in lung cancer, in vivo models inhibited tumor growth [125]. MSC can also act as an immunotherapeutic strategy by activating cellular immunity. Lentiviral transduced MSCs with T/natural killer (NK) cell-targeting chemokine CXCL9 and immunostimulatory factor OX40 ligand (OX40)/tumor necrosis factor superfamily member 4 (TNFSF4) to tumor sites improve the recruitment of CD8+ T and NK cells and reduce the autoimmunity PD-1 and MHC-1 response [126]. In addition, IFN-β expressing MSC can migrate to the 4T1 breast cancer site and secrete high levels of cytokine, which inactivates constitutive phosphorylation of the signal-transduced activator transcription factor (Stat3), Src, and Akt and downregulates cMyc and MMP2 expression [127]. MSCs expressing IFN-γ induce apoptosis in vitro in lung and breast cancer cell lines via TRAIL-mediated caspase-3 activation when co-cultured. Moreover, this treatment suppresses tumor growth in a lung carcinoma xenograft model [128].

Retroviral vectors have also been used because of their good tropism to host cells. Mo-MLV and murine stem cell virus-based vectors are used for MSC transduction [129]. Retroviral transduction also allows genetic modification of MSCs. The expression of fusion yeast CD:UPRT gene by MSC derived from adipose tissue in combination with 5-FU increases the cytotoxic effect on the colon cancer HT-29 cell line in vitro even more while inhibiting tumor growth in vivo [130]. However, nowadays, the clinical use of retroviral vectors is limited by the absence of long-term transgene expression, ineffective transduction of MSCs, and insertional mutagenesis requiring high virus doses for cell transduction [129].

In lung cancer, delivering interleukins (IL) by MSCs presented promising results. Human adipose-derived MSC lentiviral transduced with IL-12 prevented tumor growth and invasion of A549 adenocarcinoma cells [131]. IL-24 expressing MSC from the umbilical cord inhibited the growth of A549 cells in vitro and in vivo in a tumor xenograft [132]. The adenoviral replication-incompetent vector AdF35 used for transduction of MSC with IL-28A reduced OBA-LK1 viability, while it did not affect suppression in MSCs, quantified by absorbance [133].

Table 4 summarizes some applications in which the modification of mesenchymal cells with different viral systems is applied in different types of cancer.

## 5. Clinical Trials and Combination of Treatments

**Figure 2:**
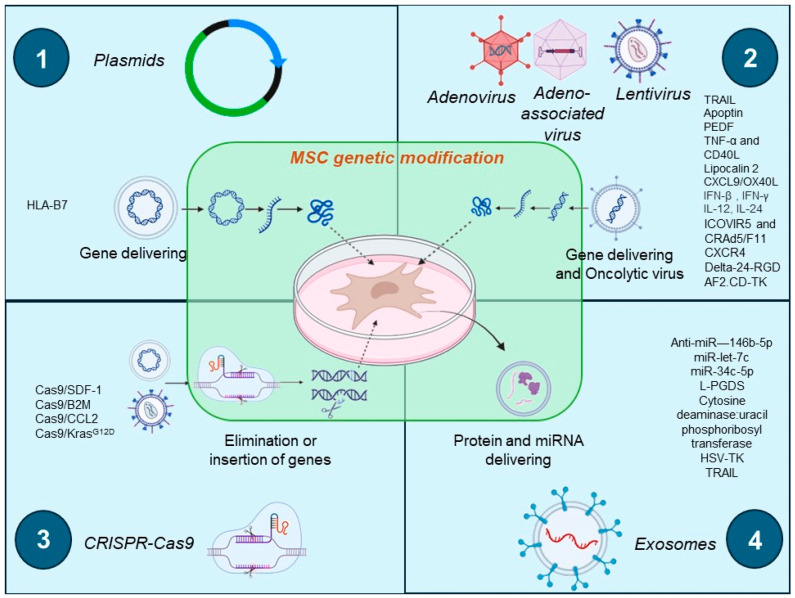
Genetic modification vectors for MSCs. 1. Plasmids in lipidic carriers for gene delivering of pro-inflammatory proteins; 2. Viral vectors of adenovirus, adeno-associated virus, and lentivirus that have oncolytic properties or gene delivery for pro-apoptotic proteins, miRNA, cytokines, and ligands; 3. Gene-editing-based techniques in MSC or tumor cells. 4. In-vitro exosome production loaded with transgene products/drugs/miRNAs.

As shown in Figure 2, both genetic modification viral and non-viral vectors are utilized in MSCs as effective carriers and delivery systems for pro-inflammatory proteins, miRNAs, enzymes, and pro-apoptotic proteins. These vectors serve as potent tools for targeted therapy against cancer. Even when MSCs have clinical potential, cancer resistance has limited their application. Thus, their combination with conventional treatment for improving delivery systems is necessary. BM-MSC-delivering therapy has been combined with chemotherapy, radiotherapy, and nanoparticles in vitro and in vivo. Table 5 includes studies of conventional therapy combined with MSC molecule delivery.

Several clinical trials have used or are using modified MSCs to evaluate their efficiency and safety to treat cancer. The study NCT02530047, phase I, used bone-marrow-derived MSC transfected with IFN-β plasmid vector by means of intraperitoneal injection in patients with ovarian cancer; by 2018, the group reported MSC engraft and INF-β expression in-situ (*n* = 3) (NCT02530047, https://clinicaltrials.gov/study/NCT02530047 (accessed on 19 June 2024)). The study NCT03298763 (TACTICAL), phase I/II, is testing MSC transduced by lentiviral vectors to express TRAIL on metastatic lung adenocarcinoma; this study is ongoing and currently recruiting patients (NCT03298763, https://clinicaltrials.gov/study/NCT03298763 (accessed on 19 June 2024)). The study NCT02068794, phase I/II is evaluating MSC infected with Edmonston’s strain measles virus that expresses sodium iodine symporter to evaluate their effect on ovarian, peritoneal, and fallopian tube cancer; this study is ongoing and currently recruiting patients (NCT02068794). The study NCT01844661 used CELYVIR, autologous MSC infected with ICOVIR5 (and oncolytic adenovirus). This approach suggests an important limitation as 18 of 19 adults could not receive the treatment as cells are of autologous origin and the disease progressed faster than the cell production; however, the study included 15 pediatric patients; results reported adenoviral replication on 13 pediatric patients and 2 patients with neuroblastoma showed disease stabilization [145] (NCT01844661, https://clinicaltrials.gov/study/NCT01844661 (accessed on 20 June 2024)); similarly, the study NCT04758533 is testing AloCELYVIR (allogeneic MSC) currently recruiting (NCT04758533, https://clinicaltrials.gov/study/NCT04758533 (accessed on 19 June 2024)). The study NCT05699811, phase I/II, aims to use MSC expressing IFN-a with or without immunochemotherapy in patients with locally advanced or metastatic cancer; this study is currently recruiting patients (NCT05699811, https://classic.clinicaltrials.gov/ct2/show/NCT05699811 (accessed on 19 June 2024)).

## 6. Perspectives

MSCs have been studied for several years as plausible cell therapy agents; however, gene delivery has emerged as a promising strategy in gene therapies and for treating various diseases. MSCs, found in different tissues of the human body, not only can differentiate into various cell types but also exhibit immunomodulatory and anti-inflammatory properties, making them valuable tools in the research and clinical application of regenerative therapies and the treatment of diseases of various origins.

Particularly in the field of cancer, MSCs have stood out for their ability to migrate to specific sites, including tumors, making them an attractive option for targeted antitumor therapies. The introduction of therapeutic genes into MSCs has led to the development of new therapies that have succeeded in inducing apoptosis, activating the immune response, and inhibiting tumor growth. However, it is crucial to identify specific targets in cancer cells to direct MSCs and avoid damaging adjacent tissues.

Various methods have been used for the genetic modification of MSCs, including viral vectors, non-viral vectors, gene editing tools, and chemical methods. Each method has its advantages and disadvantages, and the choice of method depends on the therapeutic approach, the type of cancer, and the specific goal of the therapy.

Viral vectors, such as adenoviruses, adeno-associated virus vectors (AVVs), and lentiviruses, have been widely used to transduce MSCs, offering a highly efficient method in introducing therapeutic genes. However, they have limitations, such as the possibility of oncogenic transformation and the induction of immune responses. On the other hand, non-viral vectors, such as plasmids, are less efficient but have fewer side effects. Gene editing tools, like CRISPR, allow precise editing of the MSC genome, enhancing its stemness, immunomodulatory and regenerative properties.

Despite the massive potential, CRISPR use on cancer is still limited; nevertheless, there are some applications of this technology reported on MSC. Allogeneic MSC exposed to cytokines such as IFN-γ, increase the expression of MHC class I, and this makes them easily detected by CD8+ T-cell immunity. The suppression of MHC class I in MSC by CRISPR-Cas9 RNP-mediated system to knock out the 2-microglobulin (B2M) gene, reduced MHC class I expression up to 85.1% [146]. In addition, the reduction in SDF-1 expression by CRISPR-Cas9 (MSCsSDF-1^−/−^) can be used in anti-tumor therapies to increase macrophage activation and reduce their anti-inflammatory properties [147]. CCL2 is a TAMs attractant, and currently anti-CCL2 neutralizing antibodies in mouse xenograft models prevent prostate cancer metastasis. The inhibition of CCL2 in MSC by CRISPR-Cas9 Knock out enhances MSC anti-tumor activity, with an increase in pro-inflammatory CD45+CD11b+ mononuclear myeloid cells in tumors [148].

MSC also can be used as an exosome delivery system CRISPR-Cas9. The Cas9/KrasG12D coding plasmid can be delivered by MSC to synergistic subcutaneous tumor cells to remove the DNA associated with the mutated Kras gene in tumor cells after the injection of exosomes, reducing ERK signaling and cell proliferation [147].

At the same time, chemical methods, such as nanoparticles, offer greater efficacy in gene delivery but still face challenges, such as immunogenicity and uneven distribution of nanoparticles in the tumor.

MSC homing ability has an important limitation that has been recognized since the first trials due to the fact that the majority of the transplanted cells were not able to engraft into the target tissue; however, many trials would successfully improve the patient condition despite no evidence of significant MSC integration; in the early 1990s, researchers would theorize that it was due the paracrine effect of MSCs; indeed, the majority of the cells would get caught in the capillary of the lungs but systematic communication was achieved. At present, MSC communication by means of extracellular vesicles (MSC-EXO) is well known and offers a plausible and promising cell-free therapy approach. MSC-EXO are transporters of many substances under intense research.

MSC-EXO can overcome three important MSC setbacks: first, the poor MSC engraftment, since exosomes are much smaller and exhibit homing properties as well; second, practically no risk of immune reaction; and third, no risk of cells turning malignant; additionally, exosomes can also carry products of genetic modifications, such as anti-tumor proteins, prodrugs, and miRNAs.

Many research groups have reported interesting results with MSC-EXO. In pancreatic cancer, EVs engineered with CD64 protein carrying siKRAS G12D and TP53 mRNA, silenced KRAS expression by cell cycle arrest in the G1 phase. Moreover, they suppressed orthotopic tumor growth after 2 weeks [149]. In prostate cancer, AD-MSC-derived EVs loaded with miR-145 inhibited cell proliferation and metastasis, while activating apoptosis by the Caspase 3/7 pathway [150]. Additionally in breast cancer, hBMSC EVs loaded with miR-16 inhibited angiogenesis and tumor progression [151]. Another miRNA, let-7i, delivered by EVs in lung cancer cells limited tumor cell proliferation via the KDM3A/DCLK1/FXYD3 axis [152]. Li et al. reported MSC-EVs transfected with miR-222 promoted tumor invasion and immunosuppression in colorectal tumor cells via ATF3 binding and mediation of the AKT pathway [153]. Conversely, some studies show that the cargo of MSC-EVs can inhibit the metastatic potential of tumor cells. For instance, it was demonstrated that hBMMSC-EVs loaded with miR-22-3p could suppress colorectal cell proliferation, migration, and metastasis by regulating the RAP2B and PI3K/AKT pathways [154]. Additionally, hUCMSC-EVs were found to inhibit the proliferation and migration of endometrial cancer cells by transferring miRNA-302a and downregulating the AKT signaling pathway and cyclin D1 [155]. Yao et al. identified circ_0030167, a key molecule derived from BMMSC-EVs, which inhibits the invasion, migration, proliferation, and stemness of pancreatic cancer cells by sponging miR-338-5p and targeting the Wif1/Wnt8/β-catenin axis [156]. MSC-EVs isolated from different MSC sources have been shown to either promote or suppress tumor growth, depending on their content, such as the specific miRNAs or protein cargo, which can vary under different conditions. Consequently, MSC-EVs can convey opposing signals within the same tumor type, associated with distinct subsets of miRNAs or different protein levels. However, further studies are needed to elucidate the multiple molecular signaling pathways involved in tumor growth regulation. In addition, some issues persist such as a lack of consistency in their usage in in vitro and in vivo research, as well as a need for reliable EV purification and characterization techniques. Before EV-based treatments may be used clinically, a standard method for measuring EVs delivered to cells must be developed, as well as extensive preclinical pharmacokinetic and pharmacodynamic studies. Currently, there are few clinical trials testing exosomes derived from modified MSCs; therefore, more studies are required to estimate their actual therapeutic potential.

Current evidence has demonstrated that combining conventional therapies with genetically modified MSCs and/or MSC-EXO improves treatment efficacy in in vitro and in vivo models. These studies prove that genetically modified MSCs can be a powerful tool in cancer treatment. However, despite the fact that preclinical studies are abundant, further clinical research is necessary to fully understand their mechanism of action, to optimize their therapeutic potential, and, lastly, to recognize and reduce their inherent risks.

## Figures and Tables

**Table 1:** Main therapeutic targets against solid tumors.

	Function	Target Therapy	Therapeutic Approach	References
Cancer Type				
Breast cancer		TNF-α, IL-1β, IL-6, IL-8, IFN-γ	Cytokines regulate immune system	[11]
		PI3k/AKT MYC-Max * inhibitors RTK inhibitors Anti-HER2 Anti-EGFR	Signaling pathways	[12] [13] [14] [15] [16]
		Anti-PARP	DNA repair pathway	[17]
		CPT1A/2 CYP2B6TM-RED Genes CDK4/6,	Suicide gene	[18] [19]
Colon cancer		BMP4, IL7-IL12 CX3CL NK4 Inhibitor MDM2	Immune regulatory networks	[20] [21] [22]
		TRAIL	Apoptotic proteins	[23]
		MDM2	Negative regulator of p53	[22]
Lung cancer		PD1/PDL-1 CXCL12 CXCR4	Immune regulatory networks	[24] [25,26]
		Oncolytic virus	Elimination directly	[27]
Gastric cancer		Anti-HER2 Anti-EGFR Anti-VEGF TKIs Anti-mTOR Anti-HFG/MET	Key signaling pathways	[28] [29] [30] [28]
		Anti-PARP	DNA repair pathway	[31]
Prostate		Anti-VEGFR PI3K ERK	Key signaling pathways	[32]
		Anti-CTLA-4	Immune regulatory networks	[33]
		Anti-PARP	DNA repair pathway	[34,35]
Pancreatic		HDAC inhibitors TKIs RAS-RAF-MEK-ERK PI3K-AKT-mTOR TP53	Key signaling pathways	[36] [37] [38] [39] [40]
		PARP inhibitors ATM inhibitors Checkpoint kinase 1 (CHK1) and CHK2	DNA repair pathway	[41,42] [43] [44]
		Enhance dependency on BCL-2 and/or MCL-1 inhibition)	Anti-apoptosis	[39]
Hepatocellular		Anti-HER2 GPC-3 IL-12 VEGFR GM-CSF	Key signaling pathways	[45] [46] [47] [48] [49]
		MDM2	Negative regulator of p53	[50]
		TRAIL	Apoptosis protein	[51]

* MYC/MAX heterodimer inhibition.

**Table 2:** Modified Exosomes Derived from MSCs in Cancer.

Source	Tumor Type	Approach	Reference
Umbilical cord MSC	Colorectal cancer	Exosomes loaded with Anti-miR—146b-5p ASO (PMO-146b)	[87]
Non-specified MSC	Cancer cell lines (lung, renal, breast and neuroblastoma)	Exosomes loaded with TRAIL (TNFa-Related Apoptosis Inducing Ligand).	[88]
Non-specified MSC	Gastric cancer	Exosomes loaded with lipocalin-type prostaglandin D2 synthetase (L-PGDS).	[89]
Adipose tissue MSC	Prostate cancer	Exosomes loaded with cytosine deaminase:uracil phosphoribosyl transferase along with 5-flucytosine treatment (enzyme and substrate-prodrug to synthesize 5-FU)	[90]
Adipose tissue MSC	Glioblastoma	Exosomes loaded with herpes simplex virus thymidine kinase (HSV-TK) along with ganciclovir treatment (enzyme and substrate prodrug to synthesize GCV-triphosphate)	[91]
Umbilical cord MSC	Breast cancer	Exosomes loaded with taxol.	[83]
Non-specified MSC	Breast cancer	Exosomes carrying DARPins (Designed Ankyrin Repeated Proteins) to enhance HER2+ cell uptake. Exosomes loaded with doxorubicin.	[92]
Bone marrow MSC	Castration-resistant prostate cancer	Exosomes loaded with miR-let-7c	[93]
Umbilical cord MSC	Acute myeloid leukemia	Exosomes overexpressing Lamp2b-IL3 to improve their targeting system against leukemia stem cells. Exosomes loaded with miR-34c-5p to eliminate malignant cells.	[94]
Non-specified MSC	Oral squamous cell carcinoma	Exosomes loaded with TRAIL and cabazitaxel.	[95]
Bone marrow MSC	Osteosarcoma	Exosomes loaded with doxorubicin.	[96]

**Table 3:** Main Viral Systems Used as Tools for Treating Cancer.

Virus	Ad	AVV	Lentivirus
Advantages	Low pathogenicity Safety Well-tolerated Large transgene-carrying capacity (8–36 kb) Transduce-dividing and non-dividing cells Do not integrate their genome into the host genome and remain extrachromosomal. The most common viral vectors for MSC transduction	High efficiency, safety, and lowest risk (non-inflammatory and non-pathogenic) Transgene-carrying capacity 5 kb Transduce-dividing and non-dividing cells Genome episomal (>90%) site-specific integration (<10%)	Low pathogenicity Safety Well-tolerated transgene-carrying capacity (8 kb) Transduce-dividing and non-dividing cells Integration genome High infectivity Capability of stable gene transferring
Disadvantages	Inflammatory effect	Small packaging capacity Requiring helper AdV for replication-associated difficulty producing pure viral stocks Application of these vectors has been limited due to their low aptitude for MSC transduction. Improve the efficiency of transgene delivery of Ad vectors in MSC modifications done on the viral capsid and fibers.	Transgene integration might result in oncogenesis. Next-generation lentivirus block integration into the host cell genome, and a few mutations in viral integrase coding sequence are enough to inactivate the integrase function while preserving its role in transgene expression.
References	[99,100,101,102]	[103,104,105,106]	[106,107,108]

**Table 4:** Applications of MSC Modified by Viral System.

Author	Vector	Transgene	Cancer Model	Results’ Relevance	Reference
Proteins					
Michael R. Loebinger, 2009	Lentivirus	TRAIL	Breast cancer Lung cancer	TRAIL-MSCs reduce tumor and metastasis.	[134]
Quiroz-Reyes, 2023	Lentivirus	TRAIL	Colorectal cancer	Oxaliplatin increases the sensibility of cancer cells to soluble TRAIL apoptosis.	[120]
Shahrokhi, S., 2014	Lentivirus	TNF-α and CD40L	Breast cancer	Increased mouse survival, optimized antitumor immunity response	[122]
Yan, C, 2016.	Lentivirus	ISZ-sTRAIL	Lung cancer	Apoptosis induction and tumor growth reduction in xenograft murine model	[123]
Harati, M.D, 2015	Lentivirus	Lipocalin 2	Colon cancer	Reduction of liver metastasis by downregulation of VEGF	[124]
Du, J., 2015.	Lentivirus	Apoptin	Lung cancer	Apoptosis via caspase-3 activation	[125]
Studeny, M., 2004	Adenovirus	IFN-β	Breast cancer	In situ inhibition of proliferation	[118]
Ling, X, 2010.	Lentivirus	IFN-β	Breast cancer	Inactivation of Stat3, Src, and Akt; downregulation of cMyc and MMP2 expression	[127]
Yang, X, 2014	Lentivirus	IFN-γ	Lung cancerBreast cancer	Activation of apoptosis by TRAIL-mediated caspase-3. Suppress tumor growth on a lung carcinoma xenograft.	[128].
Li, X., 2015	Lentivirus	IL-12	Lung cancer	Prevent tumor growth and invasion of A549 carcinoma cells	[131]
Zhang, X, 2012.	Lentivirus	IL-24	Lung cancer	Inhibit A549 cell growth in vitro and in vivo tumor xenograft.	[132].
Suzuki, T., 2014.	Adenovirus AdF35	IL-28A	Lung cancer	Reduction of OBA-LK1 viability.	[133].
Yin, P. et al., 2020	Lentivirus	CXCL9/OX40L	Colon cancer	Increase CD8+ T and NK cells in tumors and improve PD-1 response.	[126]
Oncolytic Virus					
Hoyos, V. et al., 2015	Oncolytic adenovirus	ICOVIR15 and Ad.iC9	Lung cancer	Increase overall survival and tumor control	[135]
Stoff-Khalili, M.A., 2007	Oncolytic adenovirus Ad5/3	CXCR4	Breast cancer	Oncolysis in MDA-MB-231 cells and reduction of lung metastasis	[113].
Guo, Y. et al., 2019	Oncolytic adenovirus	ICOVIR5	Lung cancer	Activation of T cell immunity and migration	[136]

**Table 5:** Examples of assays that used gene therapy target of MSCs in combination with conventional treatment.

Modification	MSC Delivering	Conventional Therapy	Model	Reference
Unmodified MSC	microRNA-1236	Cisplatin	In vitro	[137]
	SDF-1α/CXCR4	5-FU and doxorubicin	In vitro	[138]
Nanoparticles	Manganese oxide (MnO_2_) nanoparticles	Ce6	In vivo	[139]
	Nanoparticles	5-Fluorouracil (FU) and folinic acid (FA)	In vitro	[140]
	Nanoparticles	Paclitaxel	In vitro and in vivo	[141]
Lentiviral	TRAIL	Oxaliplatin	In vitro	[120]
Adenoviral	sFlt-1	Doxorubicin	In vitro and in vivo	[142]
Oncolytic virus	Delta-24-RGD	Chemotherapy and radiotherapy	In vivo	[143]
	AF2.CD-TK	5-FC and GCV	In vitro and in vivo	[144]
